# Supplementary material for: Seed Traits Research Is on the Rise: A Bibliometric Analysis from 1991–2020
Source: Plants (Basel). 2022 Jul 31;11(15):2006. doi: 10.3390/plants11152006 (PMC9370117; doi:10.3390/plants11152006)
Supplement: Supplementary file 1 [file plants-11-02006-s001.zip › plants-1793553-supplementary.pdf]

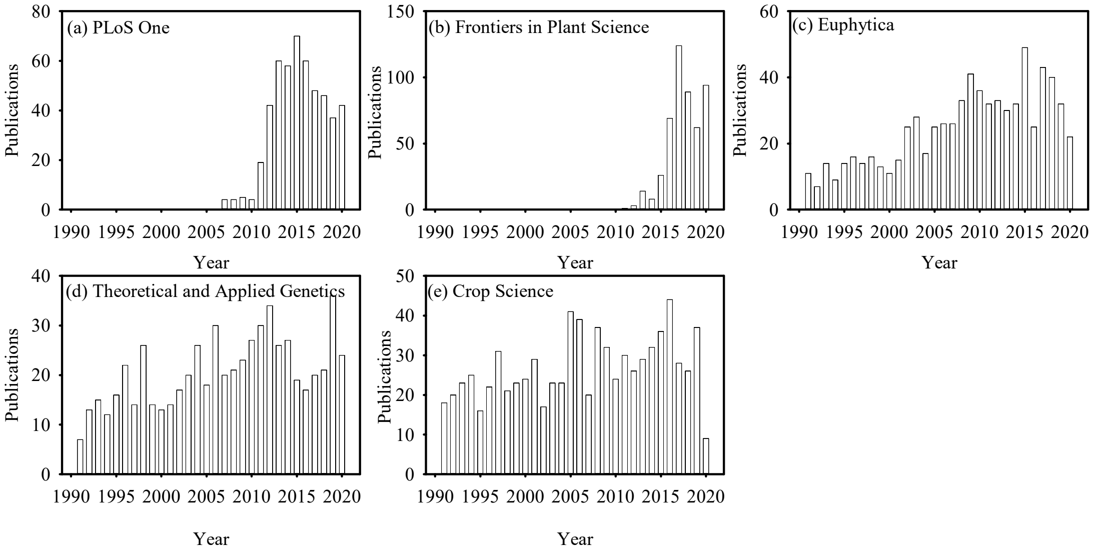

Figure S1. Time evolution trend of publications for the top five productive journals on seed traits (ST) research. (a), PLoS One; (b), Frontiers in Plant Science; (c), Euphytica; (d), Theoretical and Applied Genetics; (e), Crop Science.

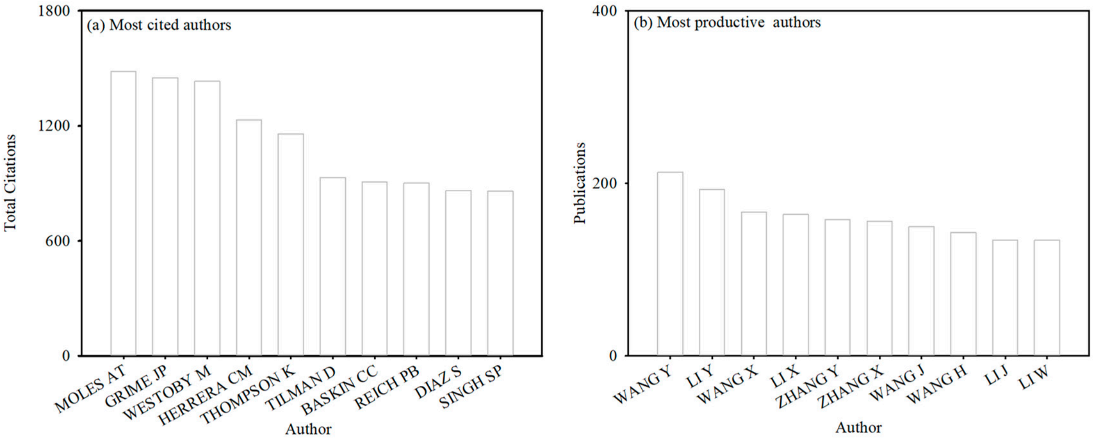

Figure S2. Top ten most cited authors (a) and most productive authors (b).

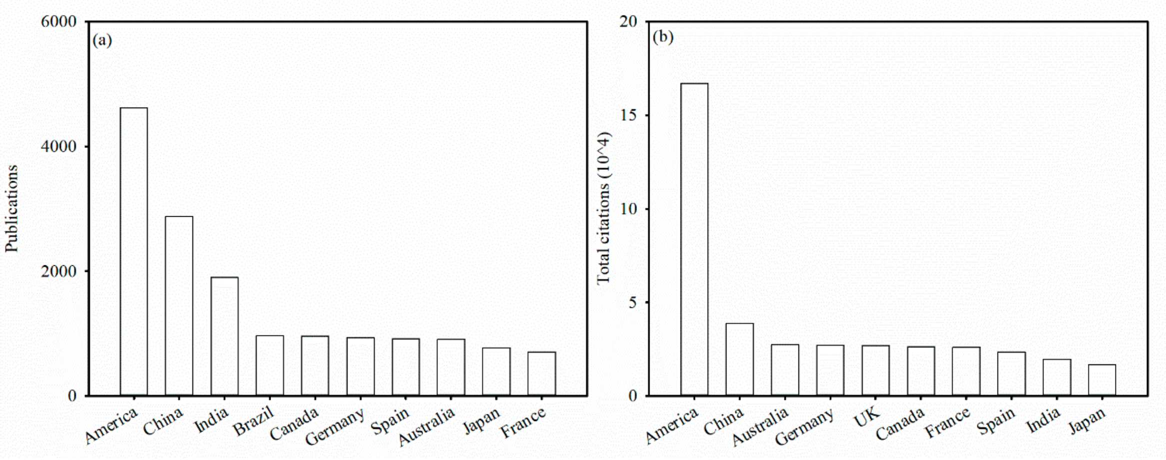

Figure S3. Top ten most relevant countries by corresponding author (a) and cited countries (b).
